# Recent methods for measuring dopamine D3 receptor occupancy *in vivo*: importance for drug development

**DOI:** 10.3389/fphar.2014.00161

**Published:** 2014-07-10

**Authors:** Bernard Le Foll, Alan A. Wilson, Ariel Graff, Isabelle Boileau, Patricia Di Ciano

**Affiliations:** ^1^Translational Addiction Research Laboratory, Campbell Family Mental Health Research Institute, Centre for Addiction and Mental HealthToronto, ON, Canada; ^2^Alcohol Research and Treatment Clinic, Addiction Medicine Services, Ambulatory Care and Structured Treatments, Centre for Addiction and Mental HealthToronto, ON, Canada; ^3^Department of Family and Community Medicine, University of TorontoToronto, ON, Canada; ^4^Department of Pharmacology, University of TorontoToronto, ON, Canada; ^5^Division of Brain and Therapeutics, Department of Psychiatry, University of TorontoToronto, ON, Canada; ^6^Institute of Medical Sciences, University of TorontoToronto, ON, Canada; ^7^Research Imaging Centre, Centre for Addiction and Mental HealthToronto, ON, Canada; ^8^Multimodal Imaging Group, Research Imaging Centre, Centre for Addiction and Mental HealthToronto, ON, Canada; ^9^Addiction Imaging Research Group, Centre for Addiction and Mental HealthToronto, ON, Canada

**Keywords:** dopamine, occupancy, PET imaging, D3, D2

## Abstract

There is considerable interest in developing highly selective dopamine (DA) D3 receptor ligands for a variety of mental health disorders. DA D3 receptors have been implicated in Parkinson’s disease, schizophrenia, anxiety, depression, and substance use disorders. The most concrete evidence suggests a role for the D3 receptor in drug-seeking behaviors. D3 receptors are a subtype of D2 receptors, and traditionally the functional role of these two receptors has been difficult to differentiate. Over the past 10–15 years a number of compounds selective for D3 over D2 receptors have been developed. However, translating these findings into clinical research has been difficult as many of these compounds cannot be used in humans. Therefore, the functional data involving the D3 receptor in drug addiction mostly comes from pre-clinical studies. Recently, with the advent of [^11^C]-(+)-PHNO, it has become possible to image D3 receptors in the human brain with increased selectivity and sensitivity. This is a significant innovation over traditional methods such as [^11^C]-raclopride that cannot differentiate between D2 and D3 receptors. The use of [^11^C]-(+)-PHNO will allow for further delineation of the role of D3 receptors. Here, we review recent evidence that the role of the D3 receptor has functional importance and is distinct from the role of the D2 receptor. We then introduce the utility of analyzing [^11^C]-(+)-PHNO binding by region of interest. This novel methodology can be used in pre-clinical and clinical approaches for the measurement of occupancy of both D3 and D2 receptors. Evidence that [^11^C]-(+)-PHNO can provide insights into the function of D3 receptors in addiction is also presented.

## INTRODUCTION

Dopamine (DA) is a neurotransmitter that has been implicated in a variety of psychiatric disorders such as Parkinson’s disease, schizophrenia, and addiction. There has been a great deal of interest in developing drugs that target DA receptors to treat these various neuro-psychiatric disorders. Five types of DA receptor subtypes have been identified and they are broadly classified as D1-type and D2-type based on sequence homology and pharmacology, and numbered in order of their date of cloning. First described in the early 1990s (Sokoloff et al., 1990), D3 receptors are a subtype of the previously characterized D2 receptor. Discovery of this subtype sparked interest in determining the properties and functions that distinguish it from D2 receptors. It is known that D3 receptors are metabotropic 7-membrane-spanning receptors that share overall ∼50% homology with the D2 receptor (Sibley and Monsma, 1992). Like D2 receptors, the D3 subtype inhibits adenylyl cyclase (Robinson and Caron, 1996). D3 receptors have been localized to neurons containing tyrosine hydroxylase suggesting that these receptors are pre-synaptic, corresponding to their role as autoreceptors (Diaz et al., 2000). This is consistent with reports that mutant mice lacking the D3 receptors are hyperactive (Xu et al., 1997), presumably due to increases in DA resulting from a lack of negative feedback normally mediated through D3 autoreceptors.

**FIGURE 1:**
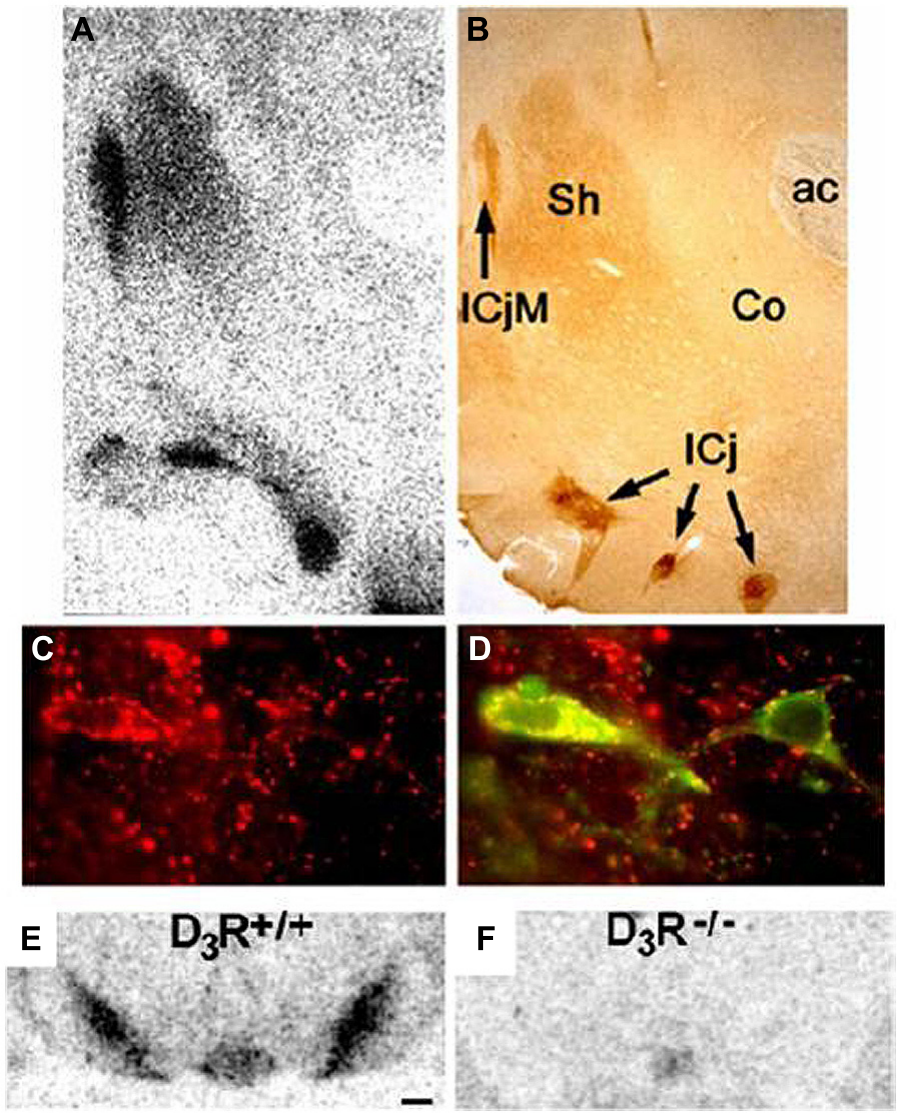
**Distribution of D3 receptors: immunohistochemical localization of the DRD3 in rat brain. (A,B)** Superimposable distributions of binding of [^125^I]*trans*-7-OH-PIPAT, a DRD3-selective ligand **(A)**, and DRD3 immunoreactivity **(B)**, with highest levels in the islands of Calleja (IcjM and IC) and moderate levels in the shell of nucleus accumbens (Sh); ac, anterior commissura. **(C,D)** Expression of DRD3 immunoreactivity alone (in red in **C**) and in combination with tyrosine hydroxylase immunoreactivity (in green in **D**). All tyrosine hydroxylase-positive neurons in the mesencephalon express the DRD3. Distribution of the binding of [^125^I]*trans*-7-OH-PIPAT in SN/VTA of D3R+/+ mice **(E)** and of D3R-/- mice **(F)**. Adapted with permission from Diaz et al. (2000).

Historically, D2 receptors have been a treatment target (mostly for schizophrenia and Parkinson’s disease), but the restricted localization of D3 receptors (Bouthenet et al., 1991; Diaz et al., 2000; Heidbreder, 2005) has led to interest in modulating D3 activity for the treatment of addiction (Le Foll et al., 2000, 2005c; Joyce and Millan, 2005), schizophrenia (Gross and Drescher, 2012), and Parkinson’s disease (Joyce, 2001; see Sokoloff et al., 2006 for a review). D3 receptors have been found to be localized to the islands of Calleja, mammillary bodies, accumbens shell, frontoparietal cortex, the substantia nigra/ventral tegmental area, and cerebellar lobules 9 and 10 (Diaz et al., 2000). As discussed later in this review, binding of radioligands to D3 receptors in the substantia nigra/ventral tegmental area is used to quantify the level of binding to D3 receptors, and **Figure 1** provides an illustration of D3 receptor immunoreactivity in the rodent brain. **Figure 1** illustrates a great density of D3 receptors in the nucleus accumbens and substantia nigra/ventral tegmental (Diaz et al., 2000).

Until recently, direct study of the D3 receptor has proven difficult due to the lack of compounds selective for D3, as opposed to D2, receptors. Nonetheless, a number of selective antagonists have been developed, including SB-277011-A (Reavill et al., 2000), YQA14 (Song et al., 2012), PG01037 (Grundt et al., 2007), NGB 2904 (Yuan et al., 1998; Robarge et al., 2001), GSK 598809 (Dodds et al., 2012; Nathan et al., 2012; Mugnaini et al., 2013), ABT-925 (Graff-Guerrero et al., 2010), ST 198 (Weber et al., 2001; Le Foll et al., 2005d), and S33138 (Millan et al., 2008). Pre-clinical studies utilizing these ligands have supported the view that DA D3 receptor antagonists may be used for the treatment of psychiatric disorders, notably addiction. Interestingly, pre-clinical findings indicate a clear relationship between *in vivo* occupancy of these receptors and behavioral response, particularly drug-seeking (see below). In human trials exploring treatment options, the measurement of occupancy of D3 receptors may thus be critical. To our knowledge, only a few human trials using a highly selective D3 antagonist (GSK 598809) have been published (Dodds et al., 2012; Nathan et al., 2012; Mugnaini et al., 2013), yet these trials have been prematurely stopped. As listed on ClinicalTrials.gov, studies examining D3 antagonists have been conducted for schizophrenia, smoking and eating disorders. Generally, clinical investigations remain in their infancy as few compounds suitable for use in humans have been developed with selectivity for D3 over D2 receptors.

A recently published review briefly summarized the [^11^C]-(+)-PHNO studies conducted in addiction to-date (Payer et al., 2014). Here, we will additionally explore the importance of differentiating the behaviors mediated by the D3 receptor from the D2 receptor. While the two receptors have historically been difficult to distinguish, their functions are distinct and therefore further investigation of D3 receptors is mandated. We will briefly introduce the published studies that indicate functional differences between D2 and D3 receptors. The present paper will also provide a more comprehensive summary of the positron emission tomography (PET) technique and PET imaging with [^11^C]-(+)-PHNO. The focus of this review will be to present novel methods allowing for the measurement of occupancy of D3 receptors in pre-clinical and clinical approaches using [^11^C]-(+)-PHNO and PET.

### THE IMPORTANCE OF DIFFERENTIATING D3 FROM D2 RECEPTORS

Despite considerable structural homogeneity, growing evidence suggests that the role of D3 and D2 receptors may be distinct. Indeed, regulation of receptor expression in various pathologies appears to differ between D3 and D2. Further, antagonists at the D2 receptor seem to be less selective in their effects on behavior which may account for the side effects observed with D2 agents but not believed to occur with D3 antagonists. D3 antagonists also have cognitive enhancing properties that are not observed with D2 antagonists (Nakajima et al., 2013). These findings are summarized below.

#### Differences between D2 and D3 receptors in pathology

Studies exploring the regulation of D2 and D3 receptors in drug addiction reveal that these receptors are differentially regulated. D2 receptors appear to be downregulated in the brains of individuals with addictions (Volkow et al., 2001). In contrast, post-mortem findings from brains of cocaine addicted individuals revealed upregulated D3 receptors (Staley and Mash, 1996). PET imaging studies in cocaine (Payer et al., 2014) and in methamphetamine polydrug users (Boileau et al., 2012; Matuskey et al., 2013) have confirmed this up-regulation. It is likely that this regulation is due to drug exposure, as various drugs of abuse, such as cocaine (Le Foll et al., 2002), methamphetamine (Le Foll et al., 2005b), nicotine (Le Foll et al., 2003), and alcohol (Leggio et al., 2014) produce this up-regulation.

With respect to schizophrenia, both D2 and D3 receptors are upregulated in post-mortem brains but the level of D3 receptors appear equivalent to controls in patients that had received antipsychotic treatment prior to death (Joyce et al., 1988). This highlights differences in the response to treatment between D2 and D3 receptors. Consistent with this, there were also no differences between controls and schizophrenics who received treatment in the binding of [125]*trans*-7-OH-PIPAT to D3 DA receptors (Gurevich et al., 1997).

In Parkinson’s disease, there is a clear up-regulation of the D2 receptor and down-regulation of the D3 receptor (Levesque et al., 1995; Morissette et al., 1998; Boileau et al., 2009). Administration of 1-methyl-4-phenyl-1,2,3,6-tetrahydropyridine (MPTP) to non-human primates produces a syndrome that resembles Parkinson’s disease (postural rigidity, bradykinesia, and akinesia) in humans and is thus used as a model of this disease. When given MPTP unilaterally, symptoms can be observed on one side of the body and neurochemical correlates can be compared to the non-lesioned side. In monkeys given unilateral MPTP, an up-regulation of D2 receptors was found in the lateral caudate and putamen (Joyce et al., 1986; Graham et al., 1990) of the treated hemisphere. This up-regulation was also found when DA levels were reduced due to 6-OHDA-induced denervation (LaHoste and Marshall, 1991). In contrast to the findings with D2 receptors, D3 receptors were down-regulated in the ipsilateral nucleus accumbens following a 6-OHDA lesion while D2 receptors were upregulated (Levesque et al., 1995). Similarly, the brains of Parkinson’s disease patients show increased D2 receptor density and decreased D3 receptors (Ryoo et al., 1998).

#### Effects on drug taking behaviors

There is considerable pre-clinical evidence suggesting that D3 receptor antagonists may be effective as treatments for addiction. Several reviews have proposed that D3 receptor antagonists may reduce relapse to the seeking of a variety of classes of drugs (Heidbreder et al., 2005; Le Foll et al., 2005c, 2007; Newman et al., 2005). D3 receptor antagonists may be especially effective in alleviating craving and relapse induced by conditioned stimuli (Le Foll et al., 2005c) in the environment and thus may block the drug-seeking induced by images and/or events associated with the drug of abuse.

The intravenous self-administration paradigm is a widely used model of drug addiction in which animals are trained to learn a new response that supplies or yields a bolus of drug. Although this model has face validity (i.e., it looks like what it supposed to measure), the effects of treatments on self-administration are not always intuitive in a simple way. Although treatments that block the reinforcing effects of drugs of abuse may decrease the amount of drug self-administered, they may also increase drug self-administration as the animal “compensates” for the reduced potency of the drug (Pickens and Thompson, 1968; Corrigall and Coen, 1989). Thus, treatment strategies that block the rewarding effects of a drug may actually lead to its increased self-administration.

These compensatory increases in behavior have been a disadvantage of D2 receptor antagonists, as opposed to D3 antagonists, in pre-clinical models. That is, blockade of the D2 DA receptor resulted in increases in responding for amphetamine (Yokel and Wise, 1975), MDMA (Brennan et al., 2009), methamphetamine (Brennan et al., 2009), or cocaine (Woolverton, 1986). By contrast, blockade of D3 receptors with selective D3 receptor antagonists had no effect on the self-administration of nicotine (Andreoli et al., 2003), cocaine (Di Ciano et al., 2003; Gal and Gyertyan, 2003; Xi et al., 2005; Achat-Mendes et al., 2010), methamphetamine (Orio et al., 2010; Higley et al., 2011), or amphetamine (Higley et al., 2012). It should be noted that one study found a decrease in the self-administration of cocaine when the animal was required to make more responses for drug (Xi et al., 2005), suggesting that instances requiring high effort to obtain drug may be affected by D3 antagonists. Further, it has been reported that D3 blockade by the selective D3 antagonist SB-277011-A decreased alcohol self-administration under low schedules of reinforcement (Thanos et al., 2005; Heidbreder et al., 2007). More recently, studies with D3 deficient mice have revealed that the D3 receptor is necessary for alcohol consumption (Leggio et al., 2014). The reason for the discrepancy between the alcohol findings and those of other drugs is unknown, but together these findings suggest that, unlike D2 receptor antagonists, D3 antagonists do not increase intake of drugs of abuse and may serve as good pharmacological treatments. In sum, the distinction between D2 and D3 receptor antagonists may be important when devising treatments for addiction.

#### Effects on locomotor activity and catalepsy

Dopamine antagonists can produce undesirable effects on locomotor activity; however, these effects are absent with D3 antagonists. Decreases in locomotor activity can be viewed as a measure of the non-specific, i.e., D2, effects of treatments and can be an indication that these antagonists will have undesired side effects. D2 antagonists have a well-known ability to reduce locomotion and induce catalepsy. By comparison, administration of D3 antagonists have no effects on spontaneous locomotion (Reavill et al., 2000; Le Foll et al., 2005a; Xi et al., 2005), stimulant-induced locomotion (Reavill et al., 2000), and are non-cataleptogenic (Vorel et al., 2002; Xi et al., 2005). Indeed, comparison of the D3 antagonist SB-277011-A to the D2 antagonist haloperidol revealed no ability to produce catalepsy of the former and significant cataleptic effects of the latter (Reavill et al., 2000). The D2 antagonist L741626 has also been observed to produce catalepsy, however, the D3 antagonists PG01037 and S33084 did so as well but to a lesser extent (Millan et al., 2000; Achat-Mendes et al., 2010). D2 antagonists blocked stimulant-induced locomotion while D3 antagonists had no effect (Millan et al., 2000). However, one study found decreases in nicotine-induced locomotion with the D3 antagonist SB-277011-A (Ross et al., 2007) used at high doses which may not be selective while another study found that the D3 antagonist NGB 2904 potentiated amphetamine-induced locomotion (Pritchard et al., 2007).

#### Selectivity of effects

Another approach to demonstrate that D3 antagonists lack non-specific effects is through evidence revealing that their effects are selective to the behavior under study. In the case of drug addiction, D3 antagonists hold promise because they seem to affect drug-relevant behaviors while sparing behaviors motivated by natural reward. This provides support not only for selectivity of effects but also makes the point that D3 antagonists, used as a treatment for drug addiction, will not have general effects on motivation. One of the most consistently reported effects of D3 antagonists is their ability to block drug-seeking behaviors in animal models of relapse (i.e., reinstatement of drug-seeking behaviors; Vorel et al., 2002; Andreoli et al., 2003; Xi et al., 2004, 2006; Gilbert et al., 2005; Gal and Gyertyan, 2006; Heidbreder et al., 2007; Achat-Mendes et al., 2010; Khaled et al., 2010; Higley et al., 2011, 2012). Importantly, this effect of reduced seeking to various drugs of abuse appears specific as no effect on food seeking behavior has been reported (Gal and Gyertyan, 2006; Xi et al., 2006; Cervo et al., 2007). Similarly, D3 antagonists are effective in blocking drug-seeking behaviors maintained by conditioned stimuli under second-order schedule of reinforcement, while producing no effects on responding for sucrose under a similar schedule of reinforcement (Di Ciano et al., 2003).

In contrast, D2 antagonists appear non-selective in their effects. That is, spiperone, haloperidol, L741626, or pimozide decreased food intake (Wise et al., 1978; Corrigall and Coen, 1991; Achat-Mendes et al., 2010). Interestingly, the latter study did not observe any effects on the first day of testing, with effects being observed only after repeated exposure of the animals to responding for food under the effects of pimozide, which suggests that D2/3 receptors are involved in the learning of this behavior. This may explain the lack of effect of eticlopride on food intake following a single treatment (Ball et al., 2011). However, eticlopride also decreased responding on a first test session when the animals were required to make a more complex response for food (Caine and Koob, 1994). Administration of haloperidol did not effect reinstatement induced by a sucrose-paired conditioned stimulus (Gal and Gyertyan, 2006), while eticlopride either increased (Ball et al., 2011) reinstatement induced by a food-paired CS or decreased it (Liu et al., 2010). Thus, D2 antagonists may be less selective and affect non-specific aspects of motivation that are absent with the highly selective D3 antagonists.

#### Effects on cognition

Evidence suggests that D3 antagonists may improve cognitive performance and thus may be viable treatments for pathologies with considerable cognitive deficits (dementia or schizophrenia; see Nakajima et al., 2013 for a review). For example, memory can be tested by imposing a delay between training and testing conditions. In the social recognition test, animals are presented with a novel juvenile rat and allowed to explore the juvenile. At a later point, the juvenile is re-introduced and exploration time of the juvenile is measured; if the animal spends less time exploring the animal than it did during the first exposure, then memory of the juvenile is intact. By increasing the delay between presentations of the juvenile, the memory for the juvenile is lost and exploratory time increases. Administration of D3 antagonists S33084 or SB-277011-A enhanced the memory for the juvenile after a delay (Millan et al., 2007).

Relative to D2 antagonists, D3 antagonists also ameliorated cognitive performance in the novel object discrimination task. In this model, time spent in exploration of a novel object is compared to exploratory time with a familiar object. Given that animals explore novel objects, they should spend more time exploring a novel object if the animal remembers the object with which it has previous exposure. Using this task, it was revealed that impairments in this task caused by a delay in the exposure and test trials were reversed by a D3 antagonist S33084. By contrast, normal performance observed without a delay was impaired by the D2 antagonist L741626 (Watson et al., 2012a). Similarly, when impairments in novel object recognition were imposed by isolation rearing, D3 antagonists also enhanced performance while D2 antagonists impaired performance under control conditions (Watson et al., 2012b). Based on these and other observations (Nakajima et al., 2013), we and others have proposed that D3 antagonists, but not D2 antagonists, may serve as cognitive enhancers.

### RECEPTOR OCCUPANCY IN ANIMALS

Pre-clinical studies have established the importance of functionally distinguishing D3 from D2 receptors. In basic pharmacological experiments, the ability of a compound to bind to D3, as opposed to D2, receptors, can be measured by studying its affinity for these different receptors. However, demonstrating that a compound will bind to D3 receptors at a given dose is essential to establish that the receptor is really occupied by the drug. Measurement of D3 receptor occupancy has been difficult due to the lack of radioligands selective for D3 over D2 receptors. The recent advent of [^11^C]-(+)-4-propyl-9-hydroxynaphthoxazine ([^11^C]-(+)-PHNO; Wilson et al., 2005), a D3 preferring agonist (Narendran et al., 2006), allows for the measurement of occupancy of D3 receptors. Specifically, the occupancy of D3 antagonists in various brain areas can be evaluated by measuring [^11^C]-(+)-PHNO binding in the presence or absence of drug. In a study by Kiss et al. (2011), [^3^H]-(+)-PHNO binding in various brain areas was antagonized by either the D3 antagonist SB-277011-A or the D2 antagonist SV-156 to determine whether binding of [^3^H]-(+)-PHNO was due to occupation of D2 or D3 receptors. They found that [^3^H]-(+)-PHNO binding in the rat cerebellar lobules 9 and 10 but not in the striatum was blocked by administration of a D3 antagonist, whereas the opposite was true for a D2 antagonist (Kiss et al., 2011). These results suggest that [^3^H]-(+)-PHNO binding in the cerebellar lobules 9 and 10 is due to D3 receptors while [^3^H]-(+)-PHNO binds to D2 receptors in the striatum. Thus, it is possible to estimate the amount of binding of a D3 antagonist by measuring occupancy of D3 receptors by [^3^H]-(+)-PHNO in cerebellar lobules 9 and 10, and conversely, to estimate occupation of D2 receptors by binding of [^3^H]-(+)-PHNO in the dorsal striatum.

This is consistent with the demonstration that SB-277011-A decreased binding of [^3^H]-(+)-PHNO in various brain areas with the greatest reduction being observed in the D3-rich substantia nigra and ventral tegmental area, and the least reduction being observed in the ventral striatum and D2-rich caudate/putamen (Rabiner et al., 2009). The binding pattern of [^3^H]-(+)-PHNO following the D2 antagonist SV-156 was complementary to that following SB-277011-A, with the most reduction in binding being observed in the dorsal striatum (Rabiner et al., 2009). Similarly, in knockout mice lacking the D3 receptor, binding of [^3^H]-(+)-PHNO was reduced in the ventral striatum and extra-striatal regions, while it was reduced in the ventral striatum and dorsal caudate-putamen in mice lacking the D2 receptor (Rabiner et al., 2009). Thus, reciprocal differences are observed in the binding of [^3^H]-(+)-PHNO in the brain. Occupancy of D3 receptors in rats can be measured by analysis of the cerebellar lobules 9 and 10 and binding of D2 receptors to the dorsal striatum.

**FIGURE 2:**
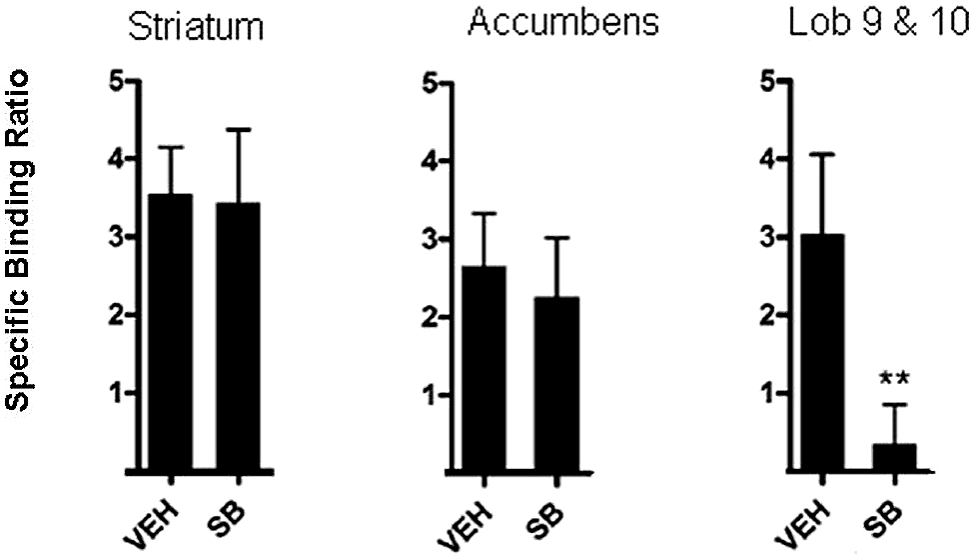
***In vivo* method for occupancy of D3 in rats.** [^3^H]-(+)-PHNO specific binding ratio in dorsal striatum, nucleus accumbens, and cerebellar lobules 9 and10 (Lob 9 & 10) after pretreatment with vehicle (veh) or the selective D3 antagonist SB-277011-A (SB). Graphs depict mean ± SD. **p< 0.01 compared to the vehicle-treated group. Adapted from McCormick et al. (2013).

Application of these principles has been used to measure the occupancy of D3 receptors by the D3 antagonist, SB-277011-A in a study by McCormick et al. (2013). To achieve this, rats were pre-treated with 10 mg/kg SB-277011-A. Sixty minutes following pre-treatment, rats were injected with [^3^H]-(+)-PHNO in the tail vein (intravenous injection) and a further 60 min allowed for uptake of the tracer. Rats were then killed by decapitation and brain areas of interest excised. Neural regions in which [^3^H]-(+)-PHNO binds to D2 receptors, namely, the dorsal striatum and nucleus accumbens, were excised. The cerebellar lobules 9 and 10, where binding is to D3 receptors, were also excised. By comparison of the binding to D3 receptors by [^3^H]-(+)-PHNO in the various brain areas it was possible to estimate the amount of occupancy of D3 receptors. As can be seen in **Figure 2**, it was reported that binding of [^3^H]-(+)-PHNO in cerebellar lobules 9 and 10 was low after administration of 10 mg/kg SB-277011-A, suggesting that this D3 antagonist occupies D3 receptors and competes with [^3^H]-(+)-PHNO which is displaced. Binding of [^3^H]-(+)-PHNO in the striatum and nucleus accumbens was high after 10 mg/kg SB-277011-A, suggesting that this compound does not readily compete with [^3^H]-(+)-PHNO for D2 receptors in these areas at this dose (McCormick et al., 2013). Collectively, these findings confirm that SB-277011-A is a D3 antagonist with high affinity for D3 receptors over D2 receptors (Reavill et al., 2000).

This approach has also been used to explore the binding of another D3 antagonist, GSK598809, in rats. In this study, binding of the radiotracer [^11^C]-(+)-PHNO to D3 receptors was decreased in humans after treatment with the D3 antagonist GSK598809 (Mugnaini et al., 2013). Notably, this study took a translational approach and demonstrated that administration of GSK598809 to rats disrupted a conditioned place preference for a nicotine-paired environment and that this ability was observed with doses which result in selective D3 occupancy (Mugnaini et al., 2013). Conditioned place preference is a model of addiction and the ability of environmental stimuli to induce approach behaviors after association with drug (Le Foll and Goldberg, 2005). This finding is consistent with the hypothesis that D3 antagonists may be especially involved in the conditioned learning of associations in addiction (Le Foll et al., 2005c).

### PET IMAGING IN HUMANS

Another method to measure occupancy of D3 receptors is through *in vivo* methods such as PET imaging in humans. PET permits the measurement of neurochemicals *in vivo* and has become a powerful tool for neuroscientists to visualize and localize receptors, measure enzymatic activity, and estimate endogenous levels of neurotransmitters (Frankle and Laruelle, 2002; Parsey and Mann, 2003; Volkow et al., 2003a,b). In PET imaging, a positron-emitting radiotracer (e.g., [^11^C]-raclopride or [^11^C]-(+)-PHNO) that binds to the protein of interest (e.g., D2 or D3 receptors), is injected intravenously and the binding of this radiotracer to receptors can be measured using the PET scanner. The D2/3 receptors were the first to be imaged using [^11^C]-*N*-methylspiperone in the living human brain using PET (Wagner et al., 1983). Since then a number of new radioligands to measure these receptors have been developed, e.g., [^18^F]-FESP, [^11^C]-raclopride, [^11^C]-*N*-methylbenperidol, [^11^C]-FLB 457, [^18^F]-fallypride (Ehrin et al., 1985; Coenen et al., 1987; Suehiro et al., 1990; Halldin et al., 1995; Mukherjee et al., 1997). Since PET imaging measures the binding of a radioligand to a receptor, changes in binding over time can be attributed to up-regulation or down-regulation of the receptor. However, PET imaging is a competitive measure, in that endogenous DA competes with the radioligand for the receptor, thereby providing an indirect measure of DA levels. The amount of binding of the radioligand is inversely proportional to the amount of neurotransmitter present such that decreases in binding of the radioligand to receptors infer an increase in DA levels.

There are two caveats to this approach: (1) although changes in DA levels can be inferred, the mechanism by which the neurotransmitter is changed is unknown. In hypothesizing whether changes are due, for example, to altered release or re-uptake, reference to pre-clinical findings must be made; and (2) since PET imaging, strictly speaking, measures binding of a radioligand to a receptor, changes in binding can be due to receptor adaptations. Thus, PET studies have within them an inherent problem of interpretation: are changes in binding potential due to altered synaptic DA or due to changes in the levels of the receptor? In general, changes in binding potential following administration of an acute challenge, for example, methylphenidate (Martinez et al., 2011), can be assumed to be related to altered DA transmission because the time frame of treatment is not long enough to observe altered regulation of the receptor. However, more long-term changes, such as those produced by chronic treatments (Brody et al., 2010), are more difficult to interpret, and parallels with the animal literature or post-mortem findings in humans can be informative. For a more detailed description of these caveats in PET imaging, see (Morris et al., 2013). Each of these approaches (measurement of DA levels and of D3 receptor levels) is considered further below.

#### Increased sensitivity in the detection of DA levels with [^11^C]-(+)-PHNO

The DA system, and in particular, D2/3 receptors, is one of the most extensively imaged receptor systems in the brain. The traditional radioligand that has been used is [^11^C]-raclopride. One limitation of PET imaging is that the sensitivity to detect changes in DA levels is low compared to that observed in animal studies, and a ceiling effect of around 40% change in receptor binding is observed (reviewed in Martinez and Narendran, 2010). Recently, [^11^C]-(+)-PHNO, a selective D3 agonist for use in PET studies with humans, has been characterized (Narendran et al., 2006; Willeit et al., 2006; Ginovart et al., 2007). Recent evidence suggests that this agonist radioligand [^11^C]-(+)-PHNO enables the detection of smaller changes in synaptic DA levels with greater sensitivity as compared to [^11^C]-raclopride. This is supported by the direct comparison of the dose-effect of amphetamine (0.1, 0.5, and 2 mg/kg; i.v.) on binding of [^11^C]-(+)-PHNO and [^11^C]-raclopride in cats (Ginovart et al., 2006). We also have recently shown enhanced ability of [^11^C]-(+)-PHNO to detect elevation of DA induced by smoking (Le Foll et al., 2014). Thus, the advent of [^11^C]-(+)-PHNO has allowed for a more sensitive measure of changes in DA levels than previously available radioligands.

#### Measurement of D3 receptors with [^11^C]-(+)-PHNO

**FIGURE 3:**
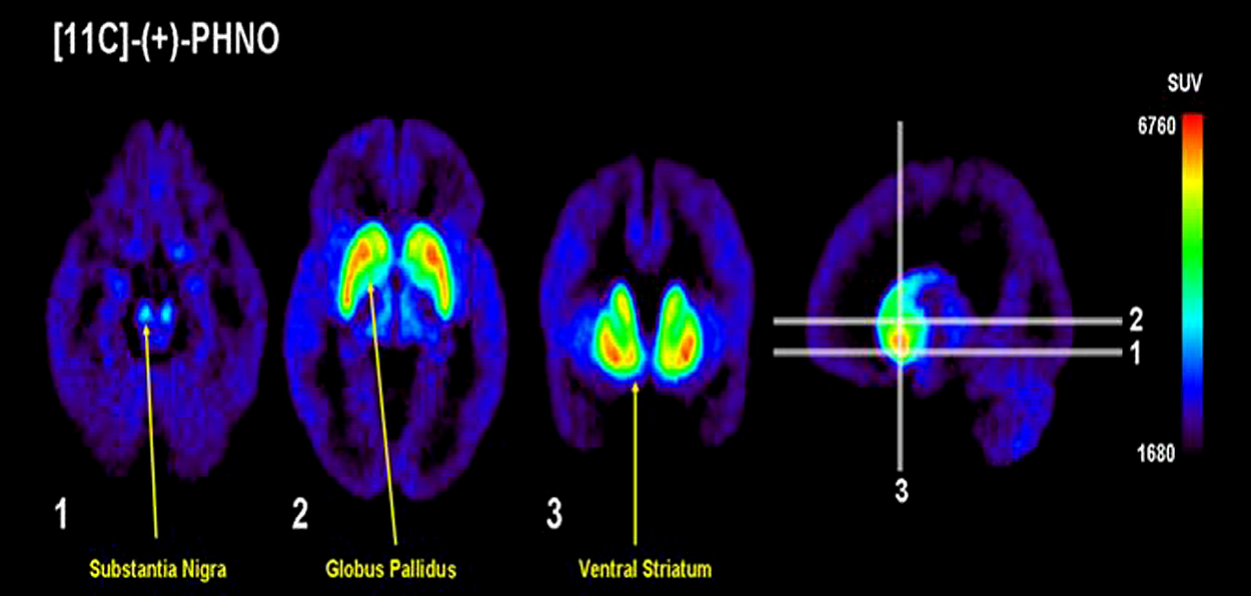
**Vizualing D3 in humans with [^**11**^C]-(+)-PHNO.** Uptake value mean images from 12 healthy controls. Note the preferential distribution in the substantia nigra, globus pallidus, and ventral striatum. Adapted with permission from Graff-Guerrero et al. (2008).

Positron emission tomography imaging of D3 receptors in humans has previously been problematic due to the lack of radiotracers selective for D3 over D2 receptors. In addition to increased sensitivity in measuring DA levels, PHNO is also more selective for D3 receptors, allowing quantification of receptor levels. Importantly however, the brain areas under investigation must be carefully considered, as the selectivity varies depending on the region of interest. D3, as compared to D2, signal is highest in the substantia nigra, hypothalamus and ventral pallidum, moderate in the globus pallidus and low/absent in the human striatum (Tziortzi et al., 2011). Consistent with the binding studies in animals, *in vivo* PET studies in humans found that [^11^C]-(+)-PHNO binding in the dorsal striatum was due to D2 receptors (Ginovart et al., 2007), while binding in the globus pallidus was due to D3 receptors [Narendran et al., 2006; see the elegant study of Tziortzi et al. (2011) for a dissection of D3 contribution to [^11^C]-(+)-PHNO signal]. [^11^C]-Raclopride, a radiotracer with equal affinity for the D2 and D3 receptors, bound more in the striatum (Graff-Guerrero et al., 2008), confirming the selectivity of binding in this region for the D2 receptor. This is further supported by the finding that [^11^C]-(+)-PHNO binding in the substantia nigra is blocked by the D3 receptor antagonist SB-277011-A in non-human primates (Rabiner et al., 2009). Thus, in estimating occupancy of D3 receptors, binding of [^11^C]-(+)-PHNO in the substantia nigra or globus pallidus can be measured, whereas [^11^C]-(+)-PHNO can also be informative as to occupancy of D2 receptors by measuring binding in the striatum. However, it should be noted that binding of [^11^C]-(+)-PHNO is not complete in all areas. Although the displacement of [^11^C]-(+)-PHNO by SB-277011-A is almost 100% in the substantia nigra and ventral tegmental area, only around 80% of the signal in the globus pallidus is attributable to the D3 receptor in mouse and baboon (Rabiner et al., 2009). This contribution is much less in the ventral striatum (around 50–60%) and even less in caudate-putamen (20–40%), consistent with the lack of selectivity of [^11^C]-(+)-PHNO for D3 receptors in these areas (Rabiner et al., 2009). **Figure 3** provides an illustration of the regional binding of [^11^C]-(+)-PHNO in the human brain. As can be seen, binding is highest in substantia nigra, globus pallidus, and ventral striatum; as such, changes in binding in these areas can reveal the degree to which a treatment is selective for D3 receptors. By comparison to areas in which [^11^C]-(+)-PHNO binding is to D2 receptors, imaging with [^11^C]-(+)-PHNO can provide a measure of the occupancy of D2 vs. D3 receptors, which is not provided by [^11^C]-raclopride.

**FIGURE 4:**
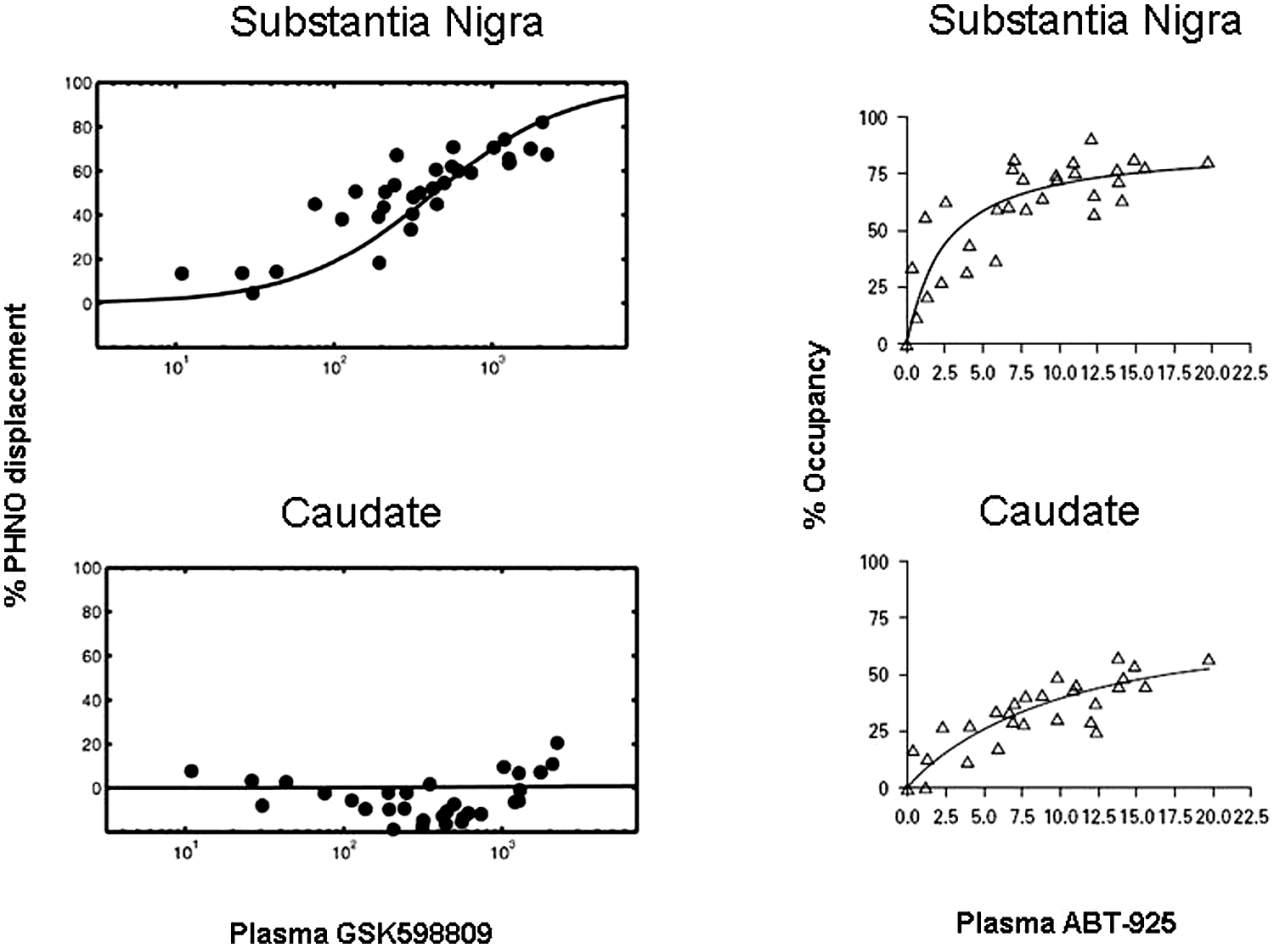
**Plots of [^**11**^C]-(+)-PHNO displacement against plasma concentration of GSK598809 or ABT-925 in two brain regions.** Relationship of [^11^C]-(+)-PHNO displacement to plasma levels of GSK598809 (left panels) and ABT-925 (right panels) in the substantia nigra (top panels) or caudate (bottom panels). At high concentrations, GSK598809 and ABT-925 almost completely blocked the specific binding of [^11^C]-(+)-PHNO in substantia nigra, with negligible effects on the caudate for GSK598809 and limited effect on the caudate for ABT-925. With permission from Searle et al. (2010) and Graff-Guerrero et al. (2010).

In a study by Searle et al. (2010), the competition of a selective D3 antagonist, GSK598809, with [^11^C]-(+)-PHNO was quantified in various brain regions and correlated with plasma levels of GSK598809. In this study, binding of [^11^C]-(+)-PHNO to receptors was expressed as displacement – the degree to which [^11^C]-(+)-PHNO binding was prevented by the antagonist. Thus, the greater the displacement of [^11^C]-(+)-PHNO, the greater the binding of GSK598809 to receptors. As can be seen in **Figure 4** (left panels), a correlation of plasma levels of GSK598809 and displacement of [^11^C]-(+)-PHNO binding to the D2/3 receptor is given. Binding of GSK598809 was greatest in the substantia nigra and also apparent in the globus pallidus, as compared to minimal binding in the ventral striatum, thalamus, dorsal caudate, and dorsal putamen. These results indicate that GSK598809 is acting primarily on the substantia nigra, then the globus pallidus and very little in the caudate, consistent with the ability to measure binding to D3 receptors [^11^C]-(+)-PHNO in the substantia nigra (Searle et al., 2010).

The ability to conduct occupancy studies with D3 antagonists in humans was also demonstrated with the administration of a selective D3 antagonist, ABT-925 (Graff-Guerrero et al., 2010). Receptor occupancy by ABT-925 was higher in the globus pallidus and substantia nigra than in caudate, putamen or ventral striatum, indicating binding of ABT-925 to D3 receptors. By comparison, ABT-925 was bound minimally in areas where binding is to D2 receptors, the ventral striatum, putamen, and caudate. ABT-925 dose-dependently bound to areas where binding is due to D3 receptors, the globus pallidus and substantia nigra, revealing the selectivity of [^11^C]-(+)-PHNO for the D3 receptor (**Figure 4**, right panels). It should be noted that these findings have been called in to question by Rabiner and Laruelle (2010), a position that has been countered (Day et al., 2010).

A recent study has been conducted with the selective D3 antagonist GSK598809 to demonstrate its binding to D3 receptors and correlate this with clinical efficacy. In this study, marked changes in binding were observed in the substantia nigra, moderate binding was observed in the globus pallidus and marginal changes were found in the dorsal striatal regions (Searle et al., 2010). Thus, binding of GSK598809 was to D3 receptors preferentially. In a subsequent study, binding of GSK598809 to D3 receptors was confirmed and extended to include behavioral tests in humans (Mugnaini et al., 2013). In that study, it was found that while under the influence of GSK598809, smokers actually increased their rate of smoking. Although this may seem contrary to the expected effects of a drug that blocks the effects of drugs, the authors propose that increases in smoking may be compensatory due to a reduced efficacy of cigarettes to deliver their reinforcing effects. As discussed above, this is not a desirable effect and one that is associated with D2 receptor antagonism in animals, not D3 receptor antagonism. However, cigarette smoking was assessed at about 8–19 h post-dose with GSK598809, and the possibility exists that a different time course of the drug administration may yield different effects. Further, clinical trials of drug efficacy for reducing smoking generally tend to assess smoking in the natural environment at several weeks during and after drug administration. Thus, further and more extensive clinical trials on the effects of D3 antagonists on drug intake are warranted, especially given the promising findings in pre-clinical studies (reviewed above).

The reasons for the termination of clinical trials with D3 agents are unknown, but it is promising that some trials showed efficacy, with one reporting a reduction in cigarette craving (Mugnaini et al., 2013), and the other reporting reductions in attentional bias to food cues in some populations (Nathan et al., 2012). These findings are tempered somewhat by further reports that, despite attenuated craving following GSK598809, cigarette smoking increased (see discussion above; Mugnaini et al., 2013) while GSK598809 did not alter brain responses to food images in obese patients (Dodds et al., 2012). These mixed findings, despite being preceded by great theoretical interest, warrant further study of D3 agents.

### ROLE OF D3 RECEPTORS: STUDIES WITH [^11^C]-(+)-PHNO

We have begun to use these imaging tools to determine the role of the D3 receptor, as compared to the D2 receptor, in addictive disorders. In our studies, we measured binding to D3 receptors using [^11^C]-(+)-PHNO and to D2 receptors using [^11^C]-raclopride, or compared [^11^C]-(+)-PHNO binding in D3-rich (substantia nigra) vs. D2-rich (striatum) areas, to determine the relative role of these receptors in addictive behaviors. To do this, we have studied not only cocaine and methamphetamine abusers, but also pathological gamblers, as gambling being recently classed as an addictive disorder in the DSM-5 (American Psychiatric Association, 2013).

In our initial study, we examined [^11^C]-(+)-PHNO binding in methamphetamine polydrug users and found that methamphetamine use was correlated with significantly higher [^11^C]-(+)-PHNO binding in the D3-rich substantia nigra as compared to healthy controls (Boileau et al., 2012). Since increased [^11^C]-(+)-PHNO binding can reflect either lower DA levels or increased number of receptors, these findings can be interpreted either way at first glance. However, pre-clinical (Le Foll et al., 2002) and post-mortem (Staley and Mash, 1996) studies have been consistent in finding increased D3 receptor number in the brains of drug-exposed individuals, suggesting that our findings with [^11^C]-(+)-PHNO reflect an up-regulation in D3 receptor number. Also consistent with established findings (Volkow et al., 2001), [^11^C]-(+)-PHNO binding in the D2-rich area of the striatum was decreased in heavy methamphetamine users. Together, the results of our study not only confirm those of past studies, but provide the first *in vivo* evidence in humans of an up-regulation of D3 receptors in addicted individuals, an effect that was opposite to that found for D2 receptors. Indeed, in a follow-up study with cocaine dependent individuals (Payer et al., 2014), participants also had increased [^11^C]-(+)-PHNO binding in the D3-rich substantia nigra as compared to controls, while [^11^C]-raclopride binding was decreased in the D2-rich striatum, as consistent with previous reports (Volkow et al., 2001). Together, these studies suggest that treatments targeting the DA system in general may not be the best strategy. That is, these approaches may produce the same response in both D2 and D3 receptors (i.e., compensatory increases in both receptor subtypes). Rather, the present findings suggest that strategies differentially affecting D2 vs. D3 receptors would be preferable. More selective approaches are needed.

Most interesting for the present discussion are findings that D3 binding in cocaine-dependent participants correlate with the number of risky choices on the Game of Dice task (a measure of risky decision making) and with errors on the Continuous Performance Task (a measure of attention and inhibitory control; Payer et al., 2014). Together, these findings implicate a relationship between D3 receptor levels and risky decision making, suggesting perhaps an addictive phenotype in that D3 receptor levels may be related to impulsivity/risky decision making. This is echoed in the additional finding that binding in D3-rich areas was correlated with motivation to use methamphetamine (Boileau et al., 2012), and, to a lesser extent, amphetamine-induced “rush,” indicating a functional relevance of up-regulation of D3 receptors.

Indeed, in pathological gamblers, we found that [^11^C]-(+)-PHNO binding in the D3-rich substantia nigra was correlated with self-reported impulsivity and severity of gambling (Boileau et al., 2013a). It should be noted that, in this study, there were no overall differences in [^11^C]-(+)-PHNO binding between pathological gamblers and healthy controls, suggesting a difference between methamphetamine and cocaine addictions and an addiction to gambling. These differences may reflect pharmacological factors related to the presence of drug in the body and receptor regulation in response to this. Further evidence for a difference between drug abusers and gamblers was found in a recent study. We demonstrated that in response to an amphetamine challenge, [^11^C]-(+)-PHNO binding in the striatum was decreased to a greater degree in the brains of gamblers compared to healthy controls, presumably due to increased DA levels (Boileau et al., 2013b). This is opposite to evidence that dopaminergic responses to challenges are blunted in the brains of drug addicts (Volkow et al., 2001; Martinez et al., 2005, 2007). As mentioned above, changes in [^11^C]-(+)-PHNO binding can reflect either receptor density or DA levels, as alterations in either will affect [^11^C]-(+)-PHNO binding. In these cited studies, since the measurements are in response to an acute challenge and under this time course it can be assumed rapid changes in [^11^C]-(+)-PHNO binding are unlikely to reflect receptor internalization. Thus these changes can be said to be due to greater increases in DA levels in pathological gamblers vs. healthy controls. In sum, gamblers have no differences in D3 receptor number as compared to controls, whereas drug addicts have upregulated D3 receptors. Further, DA eﬄux in response to a drug challenge is blunted in drug addicts, while it is augmented in gamblers. Nonetheless, the relationship of [^11^C]-(+)-PHNO to impulsiveness may be a common factor, suggesting that the correlation of D3 receptor binding to impulsiveness may highlight a phenotype susceptible to addictions. [^11^C]-(+)-PHNO has also been used to demonstrate that smoking elevates DA at the level of the D3 receptor in the human brain (Le Foll et al., 2014), an effect that confirms its relevance for nicotine addiction treatment.

## CONCLUSION

Since the cloning of the D3 receptor by Sokoloff et al. (1990), much more information is now available on its role. Pre-clinical studies have clearly delineated a role for D3 receptors in drug-seeking behavior and in motivation to take drugs. There is a clear dissociation in the functional role of D2 vs. D3 (Le Foll et al., 2009) not only for addiction, but also for other important functions such as cognition and motor control, and these findings have possible implications for treatment of schizophrenia, dementia, and Parkinson’s disease. It is therefore of foremost importance that these pre-clinical findings be translated into clinical studies. However, one caveat of previous studies has been that putative D3 ligands were used at doses that did not selectively occupy the D3 receptor (Graff-Guerrero et al., 2010). Here, we propose that the use of recently developed methods using [^3^H]-(+)-PHNO in both pre-clinical studies and human imaging studies should be incorporated. This is important in testing of highly selective D3 ligands to ensure appropriate doses are chosen. It is also useful for ligands such as buspirone, that have shown to have some D3-related effects, to determine the contribution between D2 and D3 receptors (Bergman et al., 2013; Le Foll and Boileau, 2013; Leggio et al., 2014).

## Conflict of Interest Statement

Dr. Le Foll has received grant and salary support from Pfizer Inc. and is a consultant for Richter Pharmaceuticals, Lundbeck, Mylan, Ethypharm, and Pfizer. Dr. Graff-Guerrero has received grant support from the National Institutes of Health, National Institute of Mental Health, Canadian Institutes of Health Research, Ontario Mental Health Foundation, Consejo Nacional de Ciencia y Technología, and Janssen (Johnson & Johnson).
